# Continuity of Physicians’ Dedication to Inpatient Hospice and Palliative Care: A 14-year Nationwide Survey in Taiwan

**DOI:** 10.3390/ijerph16162932

**Published:** 2019-08-15

**Authors:** Bo-Ren Cheng, Ming-Hwai Lin, Hsiao-Ting Chang, Yi-Jen Wang, Tzeng-Ji Chen, Li-Fang Chou, Shinn-Jang Hwang

**Affiliations:** 1 Department of Family Medicine, Taipei Veterans General Hospital, No. 201, Sec. 2, Shi-Pai Road, Taipei 112, Taiwan; 2 School of Medicine, National Yang-Ming University, No. 155, Sec. 2, Linong Street, Taipei 112, Taiwan; 3 Department of Primary Care and Public Health, Imperial College London, Reynolds Building, St Dunstan’s Road, London W6 8RP, UK; 4 Department of Public Finance, National Chengchi University, Taipei 116, Taiwan

**Keywords:** health workforce, hospitalization, national health programs, palliative medicine, Taiwan

## Abstract

*Background:* The work continuity of physicians in hospice and palliative medicine (HPM) has a great impact on the quality of care and practice experiences. However, nationwide studies providing a general overview of the work continuity of HPM physicians are scarce. *Methods:* Data relating to inpatient HPM care provided from July 2000 to December 2013 were obtained from the National Health Insurance Research Database of Taiwan. Specifically, the numbers of hospitals, patients, patient hospitalization days, and physicians involving HPM in each year were calculated. The years of HPM work experience and total HPM workdays of each physician were also computed. *Results:* Of the 40,965,153 inpatient records during the study, 121,258 (0.3%) records were related to inpatient HPM care, with 60 participating hospitals and 604 attending physicians. The annual number of HPM physicians increased with time from 77 in 2000 to 217 in 2013. The largest percentage (38.4%) of physicians practiced HPM for only one year, while only 23 (3.8%) physicians practiced HPM in each year without interruption. Of the 217 HPM physicians in 2013, 45 (20.7%) were newcomers, 78 (36.0%) had 1–4 years of prior HPM work experience, 54 (24.9%) had 5–9 years, and 40 (18.4%) had at least 10 years. *Conclusions:* Among HPM physicians in Taiwan, only a small percentage exhibited long-term dedication to the field, whereas most HPM physicians had short practice periods. More strategies are needed to improve work continuity among HPM physicians.

## 1. Introduction

Hospice and palliative care is a work that demands a lot of clinical experience. Physicians who dedicate themselves to hospice and palliative medicine (HPM) usually need to take care of the physical, psychosocial, and spiritual needs of patients and their families [1,2,3]. Although many studies have been conducted to improve the quality of HPM care and many efforts have been given to draw up treatment guidelines [4,5,6,7,8], an experienced physician in HPM is required to apply the results appropriately in the complex clinical situations. In order for one to become experienced, one must spend time working as a physician in HPM. Therefore, the continuity of physicians in HPM is an important issue. In the cases of studies when the continuity of physicians has been mentioned, many of them have focused on the burnout of physicians from their clinical work [9,10,11,12]. Other studies have focused on the motivation that encourages physicians working in clinical medicine [13,14]. However, nationwide studies providing a general overview of the number of years spent working in HPM and the work continuity of physicians are scarce.

The National Health Insurance (NHI) program of Taiwan is a single-payer healthcare system covering more than 99% of Taiwan’s 23 million inhabitants [15]. In July of 2000, shortly after passage of the “Hospice Palliative Care Act” in June of the same year [16], the NHI started to provide reimbursements for HPM-related inpatient care and home visits [17]. The reimbursements of HPM practices were subsequently extended to inpatient shared care [18]. The field of HPM has generally undergone successful development in Taiwan in recent years [19,20], such that Taiwan was ranked 6th in The Economist Intelligence Unit’s worldwide rankings for end-of-life care, behind only the United Kingdom, Australia, New Zealand, Ireland, and Belgium [21]. Different from Western countries, there is no nursing home or daycare unit which specifically provides HPM care services in Taiwan at present [22]. A large number of patients receiving HPM care chose a hospice ward as the place of death [22,23]. Most HPM physicians work in hospitals providing HPM inpatient care, outpatient care, and some of them providing home care.

In the current study, we examined the work continuity of HPM physicians with the provision of HPM inpatient care. We used the complete claims of the NHI program of Taiwan regarding HPM hospitalizations over 13.5 years to provide a nationwide analysis of the continuity of HPM physicians. Relatedly, this study details the characteristics of physicians in Taiwan and the duration (in years) spent working in HPM. In addition to counting the years of work in HPM for each HPM physician, we also calculated the total number of days in which each physician provided direct inpatient care within the 13.5-year period. This quantitative framework and the associated results might be of assistance in HPM policymaking and facilitate international comparisons.

## 2. Materials and Methods

### 2.1. Data Sources

The data analyzed in this study were obtained from the National Health Insurance Research Database (NHIRD), which contains extensive insurance claims for the NHI dating back to 1996 in electronic form [24]. We analyzed both inpatient claims and two kinds of registration files which contain details about medical staff and specialists. Each inpatient claim record included the patient’s identification number (ID), hospital ID, attending physician’s ID, admission date, and discharge date. Only one attending physician, usually the one attending at the time of discharge, was listed in each inpatient record. Meanwhile, the birth year, sex, and specialty of each physician were included in the aforementioned registration files. Information of daily work status and workload of HPM physicians was not recorded in the files we retrieved. All data are non-identifiable. The period examined in this study was from July 2000 to December 2013. The study was approved by the institutional review board (2013-04-005E) of Taipei Veterans General Hospital, Taipei, Taiwan.

### 2.2. Study Design

First, we extracted all of the inpatient records related to HPM. We then calculated the numbers of hospitals, patients, patient hospitalization days, and attending physicians involving inpatient HPM care in each year; for each HPM physician, we calculated the number of years during which she/he had provided care for HPM inpatients, as well as her/his total number of workdays providing inpatient HPM care. We operationally attributed the length of stay (duration between the admission and discharge dates of one hospitalization) of each patient to the attending physician, even though she/he might have had a deputy on weekends or even been on leave at some point during the hospitalization. These durations were then summed to calculate the total workdays of the given physician. We also stratified all of the physicians by their distinct years of work in HPM and then determined the distribution of HPM workdays in each physician group. We used the “distinct years” to calculate the years of work experience because the exact periods a physician worked in HPM in a year were not recorded in the files. For example, if a physician worked in HPM from December 2000 to January 2001, we would determine it as two distinct years of work experience. To illustrate the continuity of HPM work, we identified all of the physicians and the new physicians in each year and followed them until 2013. The calculation of physicians’ total workdays in HPM was in order to understand the work condition of HPM physicians (e.g., how many days do these physicians need to work in a year?). The sex, birth year, and main specialty of all the HPM physicians were also summarized.

### 2.3. Data Processing and Statistical Analysis

The programming software Perl version 5.26.2 (perl.org) was used for the data extraction, and the software Microsoft Office Excel 2016 (Microsoft Corporation, Redmond, WA, USA) was used for the statistical analysis. The results were presented with descriptive statistics.

## 3. Results

Of the 40,965,153 inpatient records of NHI claims from July 2000 to December 2013, a total of 121,258 (0.3%) records were related to inpatient HPM care, with totals of 60 hospitals and 604 physicians participating in the provision of such care. The annual numbers of hospitals and physicians increased with time. While only 16 hospitals and 77 physicians provided HPM care in 2000, those numbers had increased to 47 hospitals and 217 physicians as of 2013 (Table 1). In each year, some physicians ceased practicing and some started practicing HPM. Of the 77 HPM physicians in 2000, only 28 (36.4%) practiced HPM in 2013. On the other hand, one-fifth (45/217) of the HPM physicians in 2013 were newcomers. The annual turnover rate of the physicians who left and the ratio of the newcomers in each year were calculated and are shown in Table S1.

**Figure 1:**
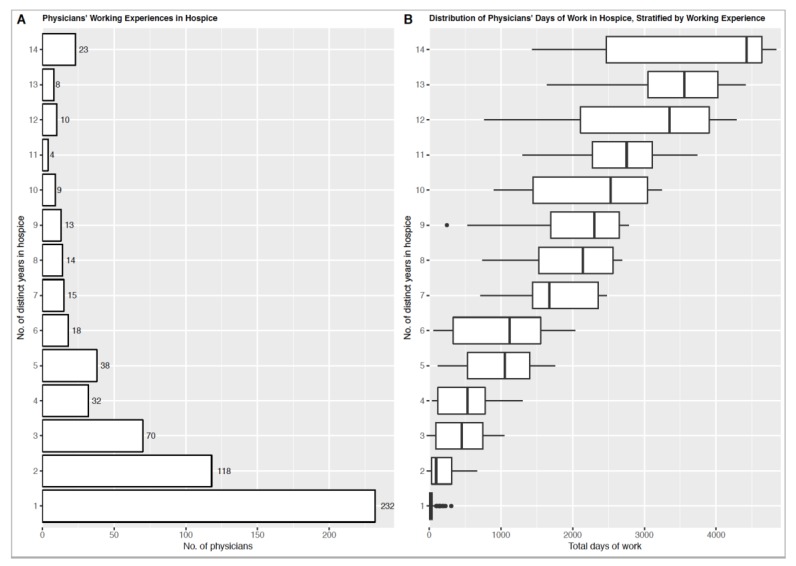
(**A**) The numbers of physicians with distinct years of HPM work experience; (**B**) The distribution of physicians’ work days, stratified by distinct years of HPM work experience.

The largest percentage (38.4%) of physicians practiced HPM for only one year (Figure 1A). Only 23 (3.8%) physicians practiced HPM in each year of the study period without interruption, while only 114 (18.9%) physicians had more than five distinct years of HPM work experience. The distribution of HPM workdays in each physician group of work experience is shown in Figure 1B. There were large variations among physicians with the same number of years of work experience. For example, among physicians with 14 years of work experience, the longest total workdays was in charge of an inpatient ward for 4839 days (average 345.6 days/year), whereas the shortest total workdays was 1436 days (average 102.6 days/year). The number of physicians who had total workdays less than one month was 189. All of them worked in HPM for less than 3 years. In 604 physicians, there were 62 physicians whose average annual workdays were more than 300 days/year; 95 physicians were between 200–300 days/year; 99 physicians were between 100–200 days/year; 118 physicians were between 30–100 days/year, and 230 physicians were less than 30 days/year. We additionally analyzed the work experience of the 217 physicians who practiced HPM in 2013: 78 (36.0%) had 1–4 years of prior HPM work experience, 54 (24.9%) had 5–9 years, and 40 (18.4%) had at least 10 years.

Among the total 604 HPM physicians in the study period, one physician’s detailed information (i.e., sex, birth year, specialty) was unknown in the registration files and was excluded from further characteristics evaluation. The overwhelming majority of the physicians were male (83.8%), and two-thirds of the physicians were born in the 1970s (34.3%) or 1960s (32.8%). Internists comprised the largest specialty group among the HPM physicians (33.3%), followed by family physicians (26.8%), surgeons (8.3%), and radiation oncologists (6.3%) (Table 2). We compared the characteristics of the HPM physicians in 2000 with those of the HPM physicians in 2013 and found an increase in the percentages of female physicians (from 10.4% to 21.2%) and family physicians (from 22.1% to 37.3%) from 2000 to 2013. Additionally, we analyzed the 28 physicians who practiced HPM both in 2000 and 2013. Most of these physicians were male (78.6%) and were born in the 1960s (50.0%) or 1950s (39.3%). Among them, family medicine (39.3%), internal medicine (28.6%), and radiation oncology (21.4%) were also the top three specialties. The characteristics of the physicians who left HPM work in 2013 and those of physicians who had ever interrupted HPM work and returned before and in 2013 are also shown in Table 2. Both of these two groups of physicians were mostly male, and the largest percentage of them were internists and born in the 1960s.

## 4. Discussion

In this study, we found that about one-third of all the HPM physicians over the entire study period were practicing inpatient HPM care in 2013, and only a small percentage (18.4%) of the HPM physicians in Taiwan in 2013 had practiced HPM for at least 10 years. There were large variations among physicians with the same number of years of work experience. Most of the HPM physicians who had practiced from 2000 to 2013 in Taiwan were male. And lastly, the main specialty of HPM physicians in Taiwan was internal medicine, followed by family medicine.

Two-third of all the HPM physicians over the entire study period left inpatient HPM care in 2013. When discussing physicians’ work continuity, burnout is always one of the important issues. Studies have shown that not only environmental factors but also personal factors can lead to burnout among HPM physicians [9,25]. Physician burnout impacts physicians’ work continuity and the maintenance of good patient care [26]. Although various studies have investigated strategies for preventing burnout [25,27], an effective solution has yet to be found.

With regard to work experiences, only a small percentage (18.4%) of the HPM physicians in Taiwan in 2013 had practiced HPM for at least 10 years. In contrast, the percentage of HPM physicians with more than 10 years of work experience in the U.S. in 2013 was 33.0% [14]. A lack of experienced HPM physicians might have impacts on the transmission of knowledge to newcomers and further influence the quality of the HPM care. Efforts should thus be made to sustain the practice of experienced HPM physicians.

For the total workdays in an inpatient ward of HPM physicians in Taiwan, the large variations among physicians with the same number of years of work experience might indicate that the working circumstances of each physician in Taiwan were quite different. Some HPM physicians might be in charge of inpatient wards throughout the year without interruption, so they would have long workdays; some might take turns with other physicians in providing care for inpatients over the course of a year, i.e., they would have worked for several months in a year; some might act as deputies when the original attending physicians were on leave, i.e., they might have worked in HPM only for a few weeks or days in a year.

Male physicians in Taiwan have always outnumbered female physicians [28], and the same phenomenon is also observed in HPM. In 2013, only 21.2% of the physicians who participated in inpatient HPM care in Taiwan were female. The gender distribution of these physicians was similar to that (female: 18.2%) for all of the physicians practicing in Taiwan in the same year [29]. In contrast with Taiwan, the percentage of female physicians in the U.S. was about 30% in 2013 [30], and 64.7% of HPM physicians in that year were female [14]. The gender distribution of HPM physicians in different cultures and medical settings thus deserves further study.

HPM is a subspecialty in Taiwan. HPM fellowship training includes direct patient care (inpatient and home care) for three months (supervised by attending physicians) and core hospice courses for 80 hours. The fellowship is available for physicians of any specialty [18,31]. In the United Kingdom, a physician takes five years to obtain a basic medical degree, and then completes two years of foundational training and six years of core medical training (including four years of palliative medicine training) to complete HPM training [32]. In the U.S., HPM fellowships are available for specific specialties (internal medicine, family medicine, physical medicine and rehabilitation, psychiatry, neurology, etc.) [33]. In this study, family physicians were found to comprise the second largest specialty group practicing inpatient HPM in Taiwan. Within the NHI program in Taiwan, a beneficiary is not required to register under a family physician, and any specialist can practice independently and see patients without referrals. On the other hand, about 40% of family physicians practice in hospitals [29], with these physicians mainly being in charge of health examinations, home care, and public health tasks in addition to outpatient consultations. These might be some of the reasons why so many family physicians were in charge of inpatient care.

There were some limitations in this study. Usually, only one attending physician’s ID could be found in each record. As such, we could not determine how many attending physicians were in charge of a patient during the course of a single hospitalization. Also, we could not find out the IDs of fellows, resident physicians, or nurse practitioners participating in inpatient care because the claims did not contain this information. A fellow would be included in our calculation as an HPM physician after he/she completed fellowship training and became an attending physician in charge of HPM inpatient ward. Furthermore, a senior HPM physician might remain dedicated to this field without providing direct inpatient care. In inpatient claims, however, such a physician would not be listed. For example, a physician might be promoted to the position of medical director and no longer provide care for inpatients. In any case, the issues above might have led to an underestimation of the number of HPM physicians.

The quality of HPM care is composed of many factors, such as the physicians’ work continuity, the quality of nursing care, the availability of psychotherapists and social workers, the provision of spiritual care, and the training of hospice volunteers. We could not analyze other factors except the physicians’ work continuity from the data we retrieved. Therefore, the quality of HPM care could not be estimated in our study. Further studies would be needed to illustrate the factors influencing the quality of HPM care.

Although we used the total workdays of each physician to indicate the workload, the actual workloads could not be calculated because of its complexity and a lack of some related factors in the database (e.g., patient disease and psychosocial complexity, time spent on different patients, mental burden, work collaboration with team members such as fellows, resident doctors, nurse practitioners). With a focus on HPM inpatient care, the provisions of HPM outpatient care and home care by physicians were not considered in this study. Such studies may be considered in the future using other corresponded claims data.

Some HPM physicians take turns with their colleagues in providing care for inpatients, so they might work in HPM for several months in a year. However, the exact years of work experience of HPM physician could not be computed because we could not determine the exact number of months or days during which a physician provided care from the inpatient claims. Therefore, we calculated the work experience of the HPM physicians by distinct years. The years of work experience for a given physician might be overestimated. For example, if a physician took his turn providing care in December of 2012 and January of 2013, he would have been viewed as having obtained two years of work experience during that time, in spite of only providing care for two months.

In our literature review, we failed to find out the studies that used nationwide datasets and calculated the HPM physicians’ years of work experience. Our study can be used for international comparison in the future when studies in other countries become available.

## 5. Conclusions

Among HPM physicians in Taiwan, only a small percentage exhibited long-term dedication to the field, whereas most HPM physicians had short practice periods. A lack of sustainable and durable workforce is of concern, and more strategies are needed to improve work continuity among HPM physicians. Besides, there were large variations among physicians in different working circumstances. Further studies would be needed to know if the variations have impacts on HPM physicians’ work continuity.

## Figures and Tables

**Table 1:** Characteristics of inpatient hospice and palliative care from 2000 to 2013 in Taiwan.

Year	No. of Hospitals	No. of Patients	Patient-Hospitalization Days	No. of Physicians in the Year	No. of Physicians Still Providing Inpatient HPM Care in 2013 (%)	No. of New Physicians in the Year	No. of New Physicians Still Providing Inpatient HPM Care in 2013 (%)
2000 ^1^	16	1970	37,423	77	28 (36.4)	77	28 (36.4)
2001	18	3919	75,345	107	33 (30.8)	48	7 (14.6)
2002	19	4532	83,302	116	43 (37.1)	44	9 (20.5)
2003	21	4768	84,473	120	47 (39.2)	38	5 (13.2)
2004	25	5336	100,077	129	51 (39.5)	33	5 (15.2)
2005	29	5759	110,410	127	64 (50.4)	37	12 (32.4)
2006	32	6769	141,871	139	66 (47.5)	32	8 (25.0)
2007	33	7261	152,972	158	81 (51.3)	44	15 (34.1)
2008	34	7515	157,839	151	93 (61.6)	30	11 (36.7)
2009	40	8180	176,139	165	109 (66.1)	39	19 (48.7)
2010	44	9281	202,487	199	119 (59.8)	52	9 (17.3)
2011	49	10,284	222,619	213	145 (68.1)	53	25 (47.2)
2012	47	10,609	228,933	200	162 (81.0)	32	19 (58.4)
2013	47	10,319	223,689	217	-	45	-
Total	60	89,243	1,997,579	604	-	604	217 (35.9)

^1^ The formal hospice and palliative care with National Health Insurance coverage in Taiwan started in July 2000. HPM = hospice and palliative medicine.

**Table 2:** Characteristics of physicians engaged in inpatient hospice and palliative care in Taiwan.

Demographic Factors	Physicians Who Ever Practiced from 2000 to 2013 (%, *n* = 603 ^1^)	Physicians Who Practiced in 2000 (%, *n* = 77)	Physicians Who Practiced in 2013 (%, *n* = 217)	Physicians Who Practiced in Both 2000 and 2013 (%, *n* = 28)	Physicians Who Left HPM Work in 2013 (%, *n* = 386 ^1^)	Physicians Who Ever Interrupted HPM Work and Returned before and in 2013 (%, *n* = 29)
Sex						
Female	97 (16.1)	8 (10.4)	46 (21.2)	6 (21.4)	51 (13.2)	4 (13.8)
Male	506 (83.9)	69 (89.6)	171 (78.8)	22 (78.6)	335 (86.8)	25 (86.2)
Birth year						
1930s	3 (0.5)	1 (1.3)	0 (0)	0 (0)	3 (0.8)	0 (0)
1940s	20 (3.3)	8 (10.4)	5 (2.3)	2 (7.1)	15 (3.9)	1 (3.4)
1950s	141 (23.4)	32 (41.6)	29 (13.4)	11 (39.3)	112 (29.0)	5 (17.2)
1960s	198 (32.8)	34 (44.2)	61 (28.1)	14 (50.0)	137 (35.5)	12 (41.4)
1970s	207 (34.3)	2 (2.6)	97 (44.7)	1 (3.6)	110 (28.5)	11 (37.9)
1980s	34 (5.6)	0 (0)	25 (11.5)	0 (0)	9 (2.3)	0 (0)
Specialty						
Internal medicine	201 (33.3)	25 (32.5)	78 (35.9)	8 (28.6)	123 (31.9)	11 (37.9)
Family medicine	162 (26.9)	17 (22.1)	81 (37.3)	11 (39.3)	81 (21.0)	9 (31.0)
Surgery	50 (8.3)	3 (3.9)	7 (3.2)	0 (0)	43 (11.1)	1 (3.4)
Radiation oncology	38 (6.3)	12 (15.6)	14 (6.5)	6 (21.4)	24 (6.2)	4 (13.8)
Others	152 (25.2)	20 (26)	44 (20.3)	3 (10.7)	115 (29.8)	4 (13.8)

^1^ One physician’s information was unknown.

## Supplementary Materials

The following are available online at https://www.mdpi.com/1660-4601/16/16/2932/s1, Table S1: The turnover rate of HPM physicians in Taiwan from 2000 to 2013.
