# Supplementary material for: Continuity of Physicians’ Dedication to Inpatient Hospice and Palliative Care: A 14-year Nationwide Survey in Taiwan
Source: Int J Environ Res Public Health. 2019 Aug 15;16(16):2932. doi: 10.3390/ijerph16162932 (PMC6720616; doi:10.3390/ijerph16162932)
Supplement: Supplementary file 1 [file ijerph-16-02932-s001.pdf]

Table S1. The turnover rate of HPM physicians in Taiwan from 2000 to 2013.

Year	No. of physicians in the year	No. of new physicians in the year	No. of physicians left in the year	Turnover rate of physicians left in the year[1]	Ratio of new physicians in the year[1]
2000	77	77	-	-	-
2001	107	48	18	19.6%	52.2%
2002	116	44	35	31.4%	39.5%
2003	120	38	34	28.8%	32.2%
2004	129	33	24	19.3%	26.5%
2005	127	37	39	30.5%	28.9%
2006	139	32	20	15.0%	24.1%
2007	158	44	25	16.8%	29.6%
2008	151	30	37	23.9%	19.4%
2009	165	39	25	15.8%	24.7%
2010	199	52	18	9.9%	28.6%
2011	213	53	39	18.9%	25.7%
2012	200	32	45	21.8%	15.5%
2013	217	45	28	13.4%	21.6%

[1]Reference: Aksu, A. Employee turnover: Calculation of turnover. In Handbook of hospitality human resources management, 2008; p 195.
